# Married

**Published:** 1870-10

**Authors:** 


### MARRIED.

WATTS—ALLEN—In Newfield, Tompkins Co., N. Y., at the residence of the bride's father, Alvah Brown, Esq., by the Rev. James Smith, Thursday afternoon, Sept. 8th, at 2 o'clock, ROBERT M. WATTS, of this city and Mrs. OLLIE B. ALLEN, of the former place.

All right, Robert. We welcome you to the ranks of the noble army of Benedicts and dub you a knight of the garter—but, doubtless that has already been done; in that case accept our congratulations and assurances that you have done the proper thing for an old bachelor to do, and that from a glimpse we have had of the bride we think you ought to be happy. If you make half as good a partner as you are a printer, your wife will indeed have found "a model husband."

